# Beyond the Concussion: Cerebral Venous Thrombosis Following Mild-to-Moderate Traumatic Brain Injury

**DOI:** 10.7759/cureus.100291

**Published:** 2025-12-28

**Authors:** Dalal Alhaffar, Paurnami Prashanth, Maryam Khan, Zaid N Herzallah

**Affiliations:** 1 Emergency Department, Rashid Hospital, Dubai, ARE

**Keywords:** cerebral venous sinus thrombosis (cvst), ct venography, dense delta sign, hemorrhagic contusions, mild traumatic brain injury (mtbi), moderate traumatic brain injury, occipital skull fracture, trauma-related cvst (tcvst)

## Abstract

Cerebral venous sinus thrombosis (CVST) accounts for a small proportion of strokes. Still, it carries meaningful clinical impact, classically linked to systemic prothrombotic conditions, hormonal influences, and severe traumatic brain injury (TBI). However, with advances in neuroimaging, it’s now evident that trauma-related CVST (tCVST) can also complicate mild to moderate head injuries even in the absence of skull fractures. We report the case of a 31-year-old South Asian male who developed tCVST following a motorbike accident. He sustained a moderate TBI with a small occipital fracture. On presentation, he was confused (GCS 14/15), and initial imaging revealed small hemorrhagic cerebral contusions. Subsequent CT venography identified thrombus formation in the torcular Herophili, extending into the superior sagittal and transverse sinuses. Given the presence of hemorrhagic lesions, anticoagulation was withheld, and the patient was managed conservatively. He showed progressive clinical improvement, with follow-up imaging demonstrating partial recanalization by day 10. He was discharged neurologically intact on day 12. This case underscores the need for high clinical suspicion of CVST across the full spectrum of TBI severity. Its clinical features may be subtle and easily misattributed to primary traumatic pathology. Early recognition, particularly through CT or MR venography, is critical for timely diagnosis. Additionally, this case highlights the likelihood that CVST is underrecognized in patients with mild TBI, reinforcing the need for prospective studies to better define its true incidence, natural history, and optimal management strategies.

## Introduction

Cerebral venous sinus thrombosis (CVST) is an uncommon but clinically significant cause of stroke, accounting for approximately 0.5-1% of cases worldwide [1]. Although it has traditionally been associated with systemic prothrombotic states, hormonal influences, and severe traumatic brain injury (TBI) with skull fractures involving venous sinuses [2], recent advances in neuroimaging have broadened its clinical spectrum [3]. Emerging evidence indicates that trauma-related CVST (tCVST) may also complicate moderate and even mild head injuries, sometimes without obvious skull fractures [4]. This evolving recognition raises an important clinical question: what is the incidence of CVST in patients with mild TBIs, and how often might these cases remain underdiagnosed due to overlapping symptoms and limited routine venous imaging? [5] In this report, we present a patient with tCVST following a motorbike accident, illustrating the need to maintain vigilance across the full spectrum of head trauma.

## Case presentation

A 31-year-old South Asian male was brought to the emergency department via ambulance following a motorbike collision. He was found sitting on the roadside at the scene, and paramedics reported that he had sustained a head injury and was initially confused. On arrival, the patient was on a stretcher with a cervical collar in place. He appeared agitated and attempted to remove the collar. His vital signs were stable with a heart rate of 90 beats per minute, blood pressure of 125/72 mmHg, respiratory rate of 18 breaths per minute, and oxygen saturation of 98% on room air. He was given 100 micrograms of intravenous fentanyl, which helped to calm him.

**Figure 1:**
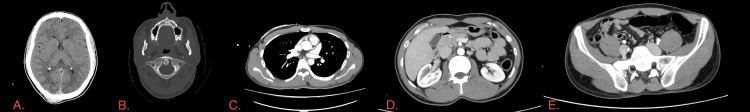
CT polytrauma showing CT of the (A) head, (B) cervical spine, (C) chest, (D) abdomen, and (E) pelvis with contrast CT: computed tomography

The patient was assessed per the ATLS protocol and had an unremarkable primary survey, except for a drop in his GCS to 14 due to confusion; the secondary survey revealed minor abrasions on the right hand. The patient also had a noticeable smell of alcohol on his breath. According to paramedic history, he had been riding a motorbike, crossed a red light, and was struck by a car. Given the mechanism of injury and altered mental status, a CT polytrauma protocol was performed, including CT of the head, cervical spine, chest, abdomen, and pelvis with contrast (Figure 1). The patient was initiated on fluid resuscitation and was given intravenous thiamine due to an unknown history of alcohol and substance abuse.

**Figure 2:**
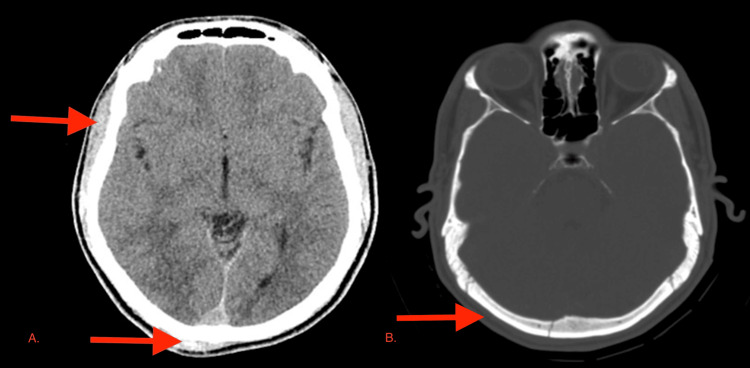
Initial CT brain showed (A) small hemorrhagic contusions in the right inferior-posterior temporal and right occipital areas and (B) non-depressed fracture of the right occipital bone CT: computed tomography

The initial CT brain showed a non-depressed fracture of the right occipital bone, along with small hemorrhagic contusions in the right inferior-posterior temporal and right occipital lobes (Figure 2)*.*

The rest of the polytrauma protocol was negative. Two hours later, the patient remained confused. Reevaluation of the brain CT revealed a dense delta sign, suggestive of venous sinus thrombosis. The case was discussed with the on-call speciality team, and the plan was to admit the patient for observation and perform CT venography (CTV) after 12 hours if no clinical improvement occurred.

**Figure 3:**
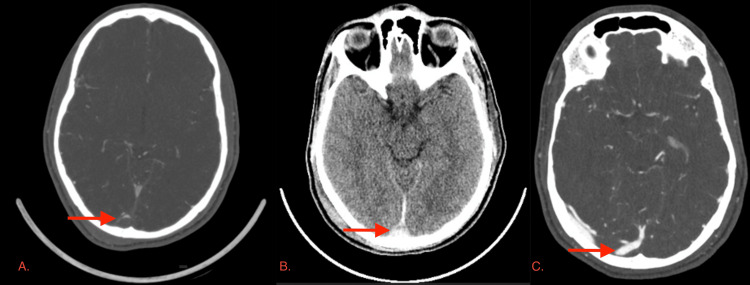
(A) CT cerebral venography showing evidence of thrombus in the torcula. (B) Follow-up CT brain demonstrated persistent hypo-density in the posterior superior sagittal sinus and right transverse sinus. (C) A repeat CTV showing only a small residual filling defect at the torcula, consistent with a small residual thrombus CT: computed tomography, CTV: computed tomography venography

In the ward, the patient remained confused with a GCS of 14/15. A CT cerebral venogram was performed, which showed evidence of thrombus in the torcula with mild extension into the superior sagittal sinus and minimal extension into the transverse sinus (Figure 3A).

The plan was to observe the patient, repeat CTV after two weeks, and obtain serial CT brain scans every two days. Anticoagulation was withheld at this stage due to the risk of worsening the brain hemorrhage.

The first follow-up CT brain demonstrated persistent hypodensity in the posterior superior sagittal sinus and right transverse sinus, as well as a thin subdural hematoma on the right tentorium (Figure 3B). There was interval regression of the hemorrhagic contusions in the right temporal and occipital lobes. After five days, the patient began to clinically improve. His GCS returned to 15/15, and he became alert and oriented to time, place, and person. His only complaint was a mild headache, which improved with mild painkillers.

A repeat CTV performed 10 days later showed only a small residual filling defect at the torcula, consistent with a small residual thrombus (Figure 3C). The remaining dural venous sinuses and cortical veins were well opacified and appeared normal.

After 12 days of admission, the patient was discharged in good neurological condition. At discharge, his GCS was 15/15, he had no focal neurological deficits, and his vital signs were stable. He was advised to maintain adequate oral hydration, undergo a repeat CTV after four to six weeks, and attend a routine neurosurgery clinic follow-up after two weeks. However, the patient failed to follow up, and he didn’t attend the clinic or repeat the CTV.

## Discussion

Epidemiology

The estimated annual incidence of CVST ranges from two to five cases per million people. However, actual rates may be higher due to increased detection through advanced neuroimaging techniques such as CTV and MR venography (MRV) [1,2]. CVST is classically associated with young females, particularly in the presence of risk factors such as pregnancy, puerperium, and oral contraceptive use, as well as systemic conditions including thrombophilia, autoimmune disease, and malignancy [1].

tCVST represents a distinct subgroup and is increasingly reported in recent years. Earlier literature suggested CVST primarily occurred in the setting of severe TBI with extensive skull fractures abutting venous sinuses. However, newer studies demonstrate that CVST can develop even after closed head injury without obvious fractures, highlighting the role of subtle venous sinus injury, endothelial disruption, or post-traumatic hypercoagulability [2]. The pooled prevalence of CVST in trauma patients with skull fractures overlying venous sinuses is estimated at 26%, with the superior sagittal, transverse, and sigmoid sinuses being the most frequently affected.

The relevance of tCVST lies not only in its occurrence but also in its associated morbidity. Recent multicenter data indicate that up to 20% of patients with post-traumatic dural venous sinus thrombosis experience unfavorable six-month outcomes [3]. Prognosis is closely related to patient age, thrombus location, extent of collateral drainage, and associated intracranial pathology. For trauma patients, the risks of hemorrhagic expansion complicate treatment decisions, making early diagnosis and tailored management essential.

Pathophysiology

CVST refers specifically to the formation of a thrombus within the dural venous sinuses, impairing venous return and leading to a cascade of complications including cerebral edema, venous infarction, and hemorrhage [1]. The broader term “cerebral venous thrombosis” encompasses thrombosis of cortical veins, deep cerebral veins, and dural venous sinuses.

tCVST is defined as sinus thrombosis occurring secondary to head injury, with or without associated fractures or hematomas [2]. While historically thought to occur only in severe injuries, tCVST has also been observed in mild-to-moderate head injury, suggesting a wider clinical spectrum.

The mechanisms underlying tCVST are multifactorial: (1) mechanical injury: fractures crossing venous sinuses can cause direct laceration, compression, or displacement of sinus walls, predisposing to thrombosis [4]; (2) external compression from epidural, subdural, or intraparenchymal hematomas, as well as cerebral edema, has been described as a mechanism of impaired venous outflow in post-traumatic CVST [5]; (3) endothelial disruption: trauma damages sinus endothelium, exposing subendothelial tissue and activating the coagulation cascade [4]; and (4) hypercoagulability: TBI is associated with systemic and localized hypercoagulable states, primarily mediated by tissue factor release, platelet activation, and inflammatory cytokines [5].

Venous obstruction increases upstream venous and capillary pressures, disrupting the blood-brain barrier, causing vasogenic edema, and reducing cerebral perfusion. In severe or unrelieved cases, cytotoxic edema and venous infarction develop, sometimes with hemorrhagic transformation [2]. Interestingly, despite these mechanisms, not all venous occlusions result in neuronal injury, due to the presence of collateral channels and spontaneous recanalization [4]. This variability explains the heterogeneous clinical outcomes observed in tCVST.

Clinical presentation

The clinical spectrum of CVST is notoriously variable, ranging from isolated headache to life-threatening intracranial hypertension. In general, approximately 80-90% of patients present with headache, often diffuse and progressive [6,7]. Up to 40% may present with seizures [6,8], and around 30-40% with focal neurological deficits [6,7].

In tCVST, distinguishing symptoms from those due to TBI itself is challenging. Both may manifest with confusion, altered mental status, focal deficits, or papilledema. In trauma patients, persistent or delayed neurological deterioration, such as worsening headache, change in sensorium, new cranial nerve deficits, or seizures, should raise suspicion for venous sinus thrombosis [2].

Our patient presented initially with confusion (GCS 14/15) following a motorbike accident and occipital fracture. While his confusion could have been attributed to contusions or alcohol intoxication, the finding of a dense delta sign and subsequent venography confirmed thrombosis of the torcula extending into the sagittal and transverse sinuses. His case illustrates the importance of maintaining a high index of suspicion, even when initial findings seem attributable to primary trauma.

Diagnosis and imaging

Non-contrast CT (NCCT) remains the first-line modality in TBI assessment but has limited sensitivity (20-43%) for CVST [4]. Direct signs include a hyperdense sinus (“cord sign” or “dense delta sign”), while indirect signs include venous infarcts, hemorrhage, or edema. For definitive diagnosis, CTV or MRV is recommended. CTV is readily available in acute trauma settings and has high sensitivity for detecting sinus thrombosis, whereas MRV offers better soft-tissue resolution and parenchymal evaluation [1]. Digital subtraction angiography remains the gold standard but is invasive and less commonly used today.

Recent studies emphasize the importance of carefully reviewing venous sinuses in all cranial trauma patients with fractures or hematomas adjacent to these structures. In one large series, 44% of patients with skull fractures near venous sinuses developed CVST, underscoring the need for vigilance [2].

In our case, the initial NCCT revealed an occipital fracture and contusions. Repeat imaging detected a dense delta sign, confirmed by CTV as torcular thrombosis with extension into the sagittal and transverse sinuses. Follow-up CTV at day 10 showed marked recanalization, consistent with the natural history of partial resolution.

Management

The management of tCVST remains controversial, primarily due to the competing risks of hemorrhage progression and thrombus propagation.

Anticoagulation

In spontaneous CVST, anticoagulation with heparin (unfractionated or low-molecular-weight) followed by oral warfarin is standard, even in the presence of small hemorrhages [3]. In tCVST, however, the role is less clear. Some studies demonstrate the safety and efficacy of anticoagulation in trauma patients, while others advocate a conservative approach when associated intracranial bleeding is present [2,4].

Surgical Management

Decompressive craniectomy may be lifesaving in cases of malignant intracranial hypertension. Hematoma evacuation or bone fragment elevation may be necessary when sinus compression is evident [5].

Endovascular Options

Thrombectomy or local thrombolysis with agents such as tissue plasminogen activator has been explored in severe cases but remains investigational in trauma settings [5].

Conservative Management

Observation with serial neuroimaging and clinical monitoring is reasonable in stable patients with limited thrombus burden and concurrent hemorrhagic lesions. This approach was adopted in our patient, leading to spontaneous improvement.

Ultimately, treatment decisions should be individualized, weighing thrombus location, clinical severity, hemorrhage risk, and available resources.

Prognosis and outcomes

The prognosis of CVST has improved significantly with early recognition and modern imaging. Overall mortality is estimated at 5-10%, with most survivors achieving favorable functional recovery [3]. However, up to 20-30% may experience residual deficits such as headache, seizures, or focal neurological impairment [1].

In tCVST, outcomes are influenced by age, thrombosis location, injury extent, and associated hematomas. Skull base fractures and involvement of the superior sagittal sinus have been linked with a worse prognosis [2]. Nevertheless, many cases show spontaneous recanalization, particularly in patients managed conservatively [4].

Our patient demonstrated gradual neurological recovery over five days, with radiological evidence of thrombus regression by day 10. He was discharged after 12 days without focal deficits and continues to do well on follow-up. His course highlights that selected cases of tCVST may resolve without anticoagulation, though vigilant monitoring remains essential.

This case highlights essential diagnostic and management lessons in tCVST. Although tCVST following mild-to-moderate TBI has been previously reported, this case underscores the diagnostic challenge posed by persistent post-traumatic confusion initially attributed to alcohol intoxication and concussion. Careful re-evaluation of routine NCCT imaging revealed a subtle dense delta sign, prompting timely CTV and diagnosis. Additionally, this case demonstrates that selected patients with tCVST and concomitant intracranial hemorrhage can be managed safely and conservatively without anticoagulation, with close clinical and radiological monitoring and spontaneous partial recanalization. These findings reinforce the need for heightened clinical suspicion and individualized management strategies in mild TBI patients with unexplained or persistent neurological symptoms.

## Conclusions

Our case underscores that CVST can occur even in the context of mild TBI, where clinical manifestations may be subtle and easily attributed to the primary trauma itself. In our patient, thrombosis of the torcula and major venous sinuses developed despite only mild neurological impairment at presentation, with subsequent spontaneous recanalization observed on follow-up imaging. This outcome highlights both the variable natural history of tCVST and the challenges of management in the setting of concomitant hemorrhagic injury.

Taken together with available literature, this case suggests that the incidence of CVST in mild TBI is likely underestimated, reflecting underdiagnosis rather than true rarity. Persistent or delayed neurological deterioration after mild head injury should prompt consideration of venous sinus thrombosis and appropriate imaging. Ultimately, early recognition and individualized management are essential to improving outcomes, while further prospective studies are needed to more accurately define the incidence of CVST in mild TBI.
